# Development and life cycle assessment (LCA) of super-oleophobic (under water) and super-hydrophilic (in-air) mesh membrane for oily water treatment

**DOI:** 10.1038/s41598-024-64803-0

**Published:** 2024-07-03

**Authors:** Umair Baig, M. Mobeen Shaukat, S. Z. Shuja, M. Asif, Nadeem A. Khan

**Affiliations:** 1 Interdisciplinary Research Center for Membranes and Water Security, King Fahd University of Petroleum and Minerals, 31261 Dhahran, Saudi Arabia; 2 Department of Mechanical Engineering, King Fahd University of Petroleum and Minerals, 31261 Dhahran, Saudi Arabia; 3 Interdisciplinary Research Center for Renewable Energy and Power Systems, King Fahd University of Petroleum and Minerals, 31261 Dhahran, Saudi Arabia; 4 Department of Architectural Engineering, King Fahd University of Petroleum and Minerals, 31261 Dhahran, Saudi Arabia

**Keywords:** Mesh membrane, Titania nanoparticles, Coatings, Super-hydrophilic surface, Super-oloeophobic underwater, Environmental impact, Life cycle as-assessment, Environmental sciences, Engineering

## Abstract

This paper reports the fabrication, characterization, and environmental impact analysis of a super-oleophobic (under water) and super-hydrophilic mesh membrane for oily water treatment. In order to prepare mesh membrane, Titania nanoparticles (NPs) were spray coated on mesh stainless steel followed by calcination at 500 °C. After that, the Titania-coated mesh membrane was characterized using contact angle goniometry (CA), XRD, FE-SEM, EDX and elemental mapping. The FE-SEM, EDX, elemental mapping and XRD results confirmed that the Titania NPs were successfully coated on the surface of mesh membrane. CA results demonstrated that the prepared mesh membrane is super-hydrophilic and super-oleo phobic under water conditions, making it suitable for oil/water separation. Subsequently, life cycle assessment (LCA) was performed to determine the environmental impacts of Titania NPs-coated mesh membrane fabrication process. LCA results indicate that electricity and nitrogen contributed the most toward the eighteen environmental impact categories considered for this study.

## Introduction

Mixing of oil in water can happen by various inadvertent and intended ways such as leakage from oil tankers, waste oil release into water bodies, and during oil excavation that brings about enormous quantity of oily water^1,2^, and this oil contamination in water bodies poses substantial environmental repercussions, particularly to marine life^3^. Considering the impending vulnerability to the ecosystem, there is growing global awareness to tackle this issue, and as a result, individual countries and world organizations have implemented strict regulations to control such environmental manipulation^4^. During the mining of oil, the production of oily water from oil and gas industry is unavoidable, as large amounts of water needs to be poured into oil wells, especially aging ones, to enhance oil extraction^5^. Oil companies are forced to adhere to certain stipulations to protect the delicate environment and also to recover reusable water. For oil/water separation, a wide range of technologies have been developed, including magnetic separation, chemical separation, centrifugation, filtration, flotation, and oil absorbents.^6–8^. It should be noted that in addition to the oil contamination, water may get polluted by organic/inorganic substances and micro-organisms that require purification after oil–water separation^9,10^. In recent years, surface wettability-based separation membrane for the water remediation has proven to be very successful.

The wettability of a membrane surface depends on the relative magnitudes of interfacial surface energy and surface tension of a liquid. If the surface energy exceeds the liquid's surface tension, wetting occurs, whereas dominant liquid surface tension results in beading on the surface. Various filtering systems utilizing specially designed membranes have been developed for oil–water separation^6–8,11^. However, these systems often suffer from rapid membrane pore clogging by oil or oily water, leading to a decline in their performance. Membranes with preferential wettability offer a potential solution to this problem^12–15^. By controlling the chemical composition of coatings and surface roughness, desired wettability can be achieved. Some research groups have successfully prepared membrane surfaces that exhibit a high affinity for water while repelling oil^16,17^. The membranes with super-hydrophobic and super-oleophilic properties, which allow oil to permeate through their pores due to strong oil affinity, can achieve separation efficiencies close to 95%. However, pore-clogging still remains as a technical challenge that needs to be addressed. In order to overcome oil clogging issue, membranes with high affinity for water and strong repulsion for oil (super-hydrophilic and super-oleophobic) were developed^6–8^. As the surface tension of water is significantly higher than that of oil, fabricating membranes with the above combination of surface wettability is challenging.

By selecting a material with appropriate surface energy and surface roughness, a super-hydrophilic (in-air) membrane surface that switches to super-oleophobic under the water can be realized^14,15,18^. The filtering mechanism of these kinds of membrane is that, due to the newly achieved enhanced under water oleophobicity in conjunction with right pore size, water is pushed downward by the negative downward water pressure, while the oil is pushed away from the membrane by the positive upward oil pressure. Since membrane is water passing kind, the oil clogging in the pores of the membrane is significantly reduced that leads to the stability and reusability of the membrane. Several materials have been tried to achieve this phenomenon including but not limited to SiO_2_, Co_3_O_4_, WO_3_ etc.^18^. Some recent reports indicated that certain polymeric materials exhibit super-hydrophilic/super-oleophilic wetting behavior, but the limitation of such material in the context of oil water separation is the lack of durability. In this study, based on our previous findings^19^, Titania (TiO_2_) nanoparticles (NP_s_) were chosen as a coating material for the fabrication of super-oleophobic (under water) and super-hydrophilic (in-air) mesh membrane due to its exceptional super-hydrophilicity, high stability, cheap cost, and commercial availability, in order to determine the environmental impacts of Titania NPs-coated mesh membrane fabrication process using life cycle assessment (LCA).

The environmental impact of new industrial methods and processes must be investigated in order to lessen the growing threat posed by climate change. Before new materials and methods created in the lab are suitable for mass production, their effects on the environment are typically not thoroughly investigated. However, reducing the negative effects of these activities on the environment can be achieved by looking into them. One of the most popular techniques for assessing how systems, processes, and products may affect the environment is life cycle assessment, or LCA. It has been frequently used to assess the sustainability of processes and systems in industrial and research settings for the past thirty years or so^20,21^. The environmental effects of a product's life cycle, including its extraction from the ground, transportation, production, recycling, and other stages, can be determined with the aid of life cycle assessment (LCA). The phases that have a detrimental influence on the environment are highlighted through the application of LCA. These factors have led to the application of LCA in a number of research papers to report environmental impacts. Tian et al.^22^, for instance, employed life cycle assessment (LCA) to assess the environmental effects of different solar cell recycling strategies. In the same way, Rubin ^23^ has employed it to ascertain the effects of circuit board recycling on the environment. This paper aims to assess the environmental effects of the SSM coating process by Life Cycle Assessment (LCA). This will assist in highlighting possible causes of these effects.

This paper presents the fabrication of a surface engineered mesh membrane by spray coating Titania NPs. The fabricated mesh membrane was super-hydrophilic in the air and super-oleo phobic under water. This membrane can be used for the treatment of oil-contaminated water and oil–water mixture/emulsion separation applications. LCA was conducted to determine the environmental impacts of the fabrication process. LCA takes a holistic approach to determine the environmental impacts of all stages of a process. By analyzing the entire process, LCA helps us to identify environmental hot spots-stages that contribute the most towards various environmental impact categories such as greenhouse gas emissions, resource depletion, acidification etc. As environmental regulations become more stringent, LCA can provide accurate data for compliance with relevant environmental standards. Using LCA during lab stage development of novel chemical processes, help in early identification of environmental hot spots, comparison of alternative mechanisms, and provide support for green chemistry.

## Experimental methods

### Mesh membrane fabrication and characterization

**Figure 1:**
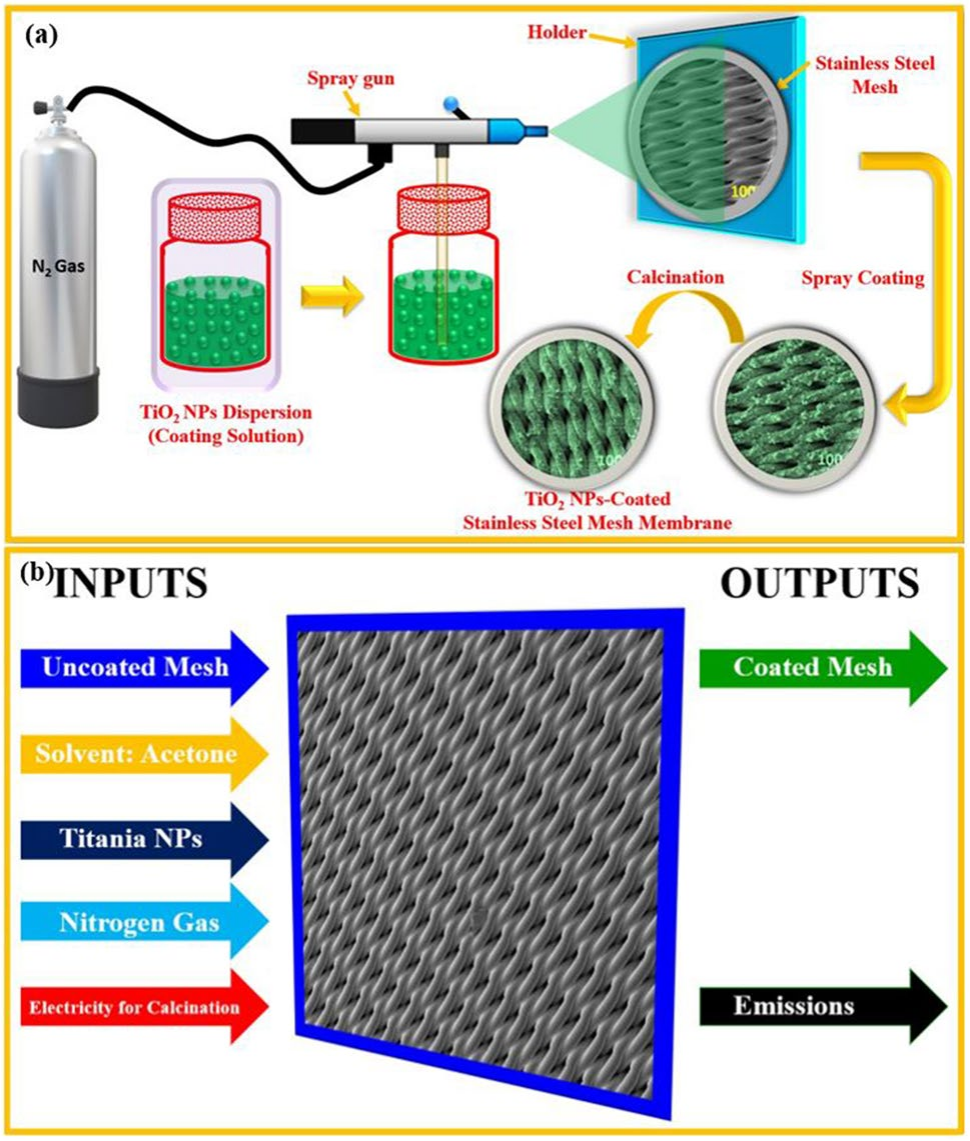
(**a**) Graphical illustration for the fabrication of Titania NPs-coated mesh membrane using simple spray-coating and calcination process. (**b**) System boundary for cradle-to-gate LCA for Titania NPs-coated mesh membrane.

The Titania NPs-coated mesh membrane was fabricated by the same method as described in our previous study^19^. The mesh (stainless-steel; SS) used for this study was acquired from TWP Inc USA and Titania NPs (particle size 10-20 nm) were acquired from Sigma Aldrich (USA). In order to prepare the mesh membrane, first the solution (in acetone) of Titania NPs was subjected to sonication. Sonication helps to clear out the lumps and make the solution homogeneous. The next step was to clean mesh using alkaline bath and distilled water. The cleaned mesh was then calcined at 300 °C for 4 h. The calcination process results in a rough surface of the mesh. Next, the Titania NPs were sprayed on the mesh surface. After that, the coated mesh was calcined again for four hours at 500 °C. Figure 1a delineates the fabrication steps used in this study. After the preparation of the mesh membrane, XRD analysis was performed by Rigaku XRD. A FE-SEM (TESCAN) was used to analyze the surface morphology and elemental mapping. Surface wettability of the mesh membrane was measured for water and oil using contact angle goniometer (KRUSS). Atomic force microscopic (AFM) analysis of the mesh membranes carried out by using Agilent 5500 AFM. The oil–water separation performance of Titania NPs-coated mesh membrane was evaluated using as per our previous studies^12,13^.

### Life cycle assessment

For this paper, LCA was conducted by following ISO 14040 standard. In accordance with this standard LCA study consists of four steps: defining the goal and scope of a study, compilation of life cycle inventory, life cycle impact evaluation, and interpretation of the results^24^. The objective of this LCA study was to quantify the environmental impacts of lab scale production process for a mesh membrane coated with Titania NPs. The functional unit for this study was defined as the production of 6 cm × 6 cm piece of Titania NPs-coated mesh membrane. Figure 1b illustrates the system boundaries for this LCA study, and Table 1 lists energy and materials consumed for this process. These values were used for conducting LCA. As the focus of this study was the process of producing Titania NPs-coated mesh membranes, a cradle-to-gate LCA approach was adopted. This LCA considers the life cycle of a product or process from resource extraction (cradle) to the factory gate before the product is shipped to consumers. LCA was performed using SimaPro software^25^, which is widely recognized as one of the best programs to conduct LCA studies. The 18 midpoint environmental consequences were calculated using the ReCiPe impact assessment approach. ReCiPe method was chosen since it is an internationally recognized method and has been utilized in several life cycle impact studies^26^.

**Table 1:** LCA data.

Material/energy	Quantity	Ecoinvent unit process
Acetone	0.00784 kg	Acetone, liquid {RoW}|market for acetone, liquid|APOS, S
Stainless steel mesh	0.0015 kg	Steel wire rod/GLO
Titanium dioxide nanoparticles (Titania)	0.0001 kg	Titanium dioxide {RoW}|market for|APOS, S
Nitrogen gas (pressure of 170 kPa)	0.116 kg	Nitrogen, liquid {RoW}|market for|APOS, S
Electric heating furnace	0.16 kWh	Electricity, high voltage {SA}|market for|APOS, S

## Results and discussion

### Structural, morphological and particle size analysis of Titania NPs

**Figure 2:**
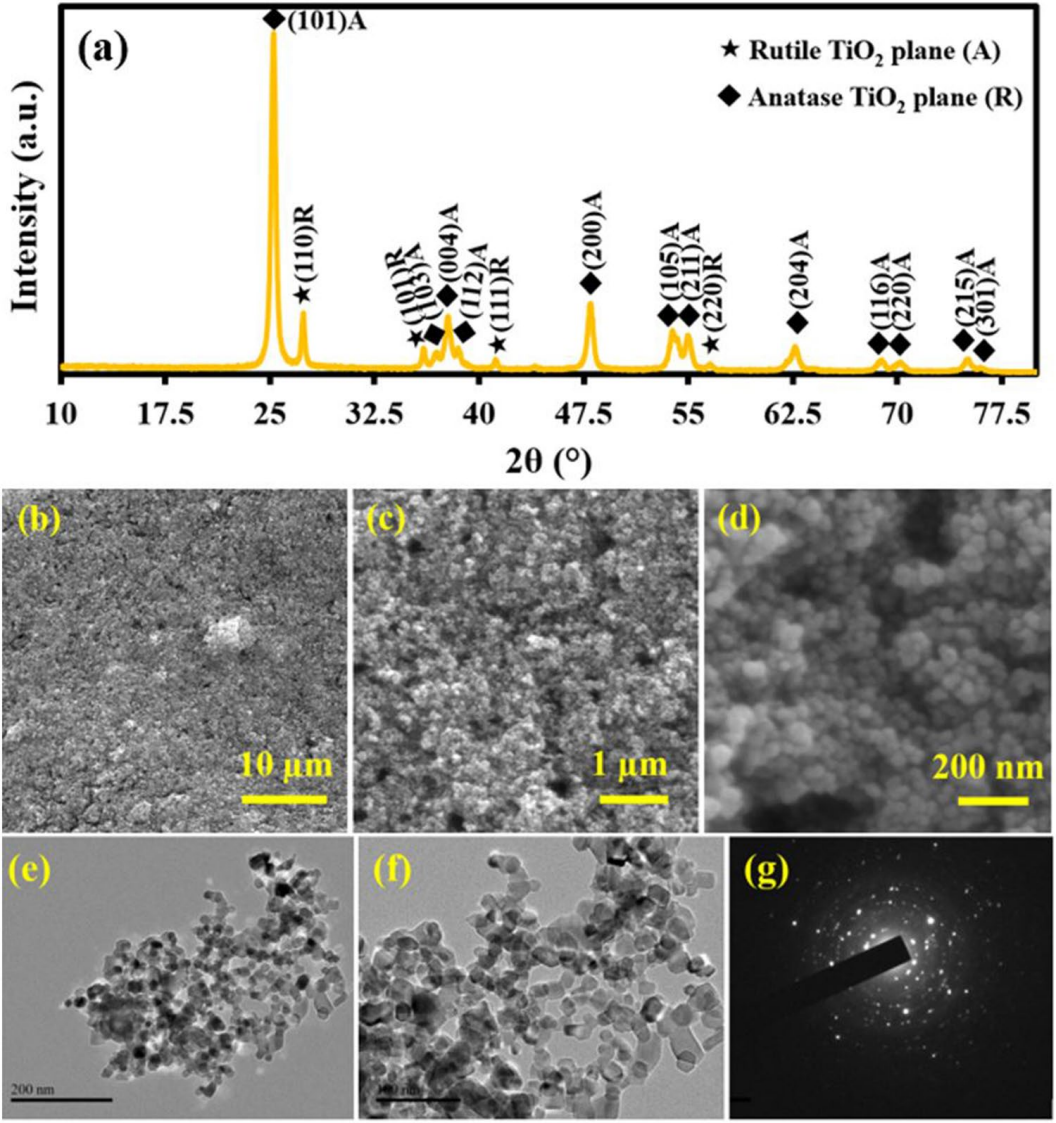
XRD pattern of Titania NPs (**a**), FE-SEM images of Titania NPs at different magnification scales (**b**–**d**) and TEM (**e**,**f**) and SAED (**g**) images of Titania NPs.

**Figure 3:**
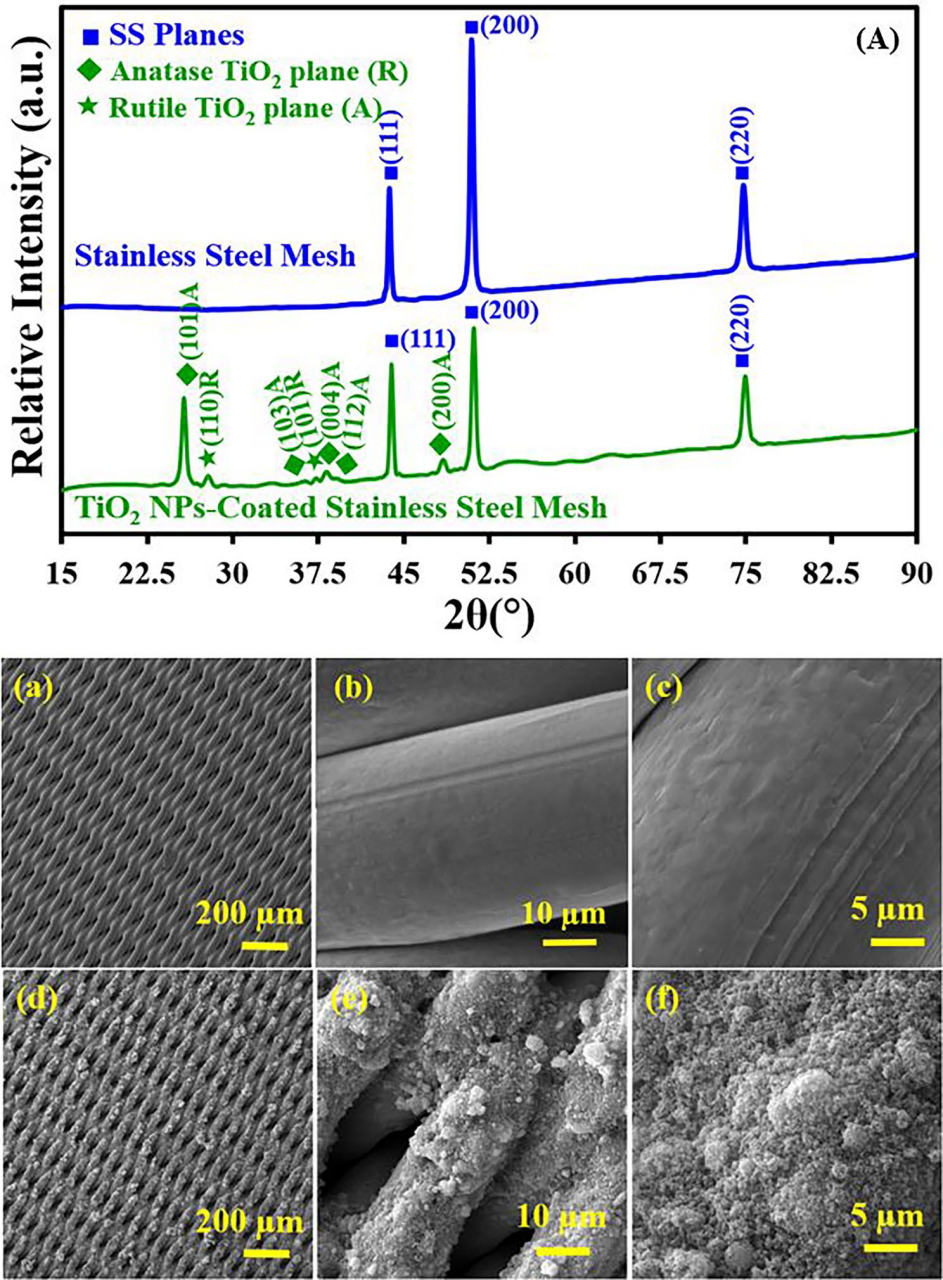
XRD patterns of bare mesh and Titania NPs-coated mesh membrane (A). FE-SEM images of bare mesh (**a**–**c**) and Titania NPs-coated mesh membrane (**d**–**f**) at different magnification scale.

The crystal structure, morphology and particle size of the Titania NPs were evaluated using XRD, FE-SEM and TEM analysis. Figure 2a depicts XRD pattern (2θ range from 10 to 80) of pure Titania NPs. In Fig. 3a, the characteristic diffraction planes are present for anatase and rutile phase of Titania. The diffraction planes (101)A, (103)A, (004)A, (112)A, (200)A, (105)A, (211)A, (204)A, (116)A, (220)A, (215)A and (301)A are pertain to the anatase phase of Titania and (110)R, (101)R, (111)R, (220)R diffraction planes due to of rutile phase of Titania are present. These diffraction planes are indicating the crystal structure of the Titania NPs having both phases anatase and rutile. FE-SEM images of Titania NPs at different magnifications are shown in Fig. 2b–d. From the FE-SEM images of the Titania NPs, it is clear that the Titania NPs have spherical shape and has highly agglomerated nature. TEM images Titania NPs at different magnifications are also shown in Fig. 2e,f. From the TEM images of the Titania NPs, it is also clear that the Titania NPs have spherical in nature and having 10–20 nm particle size. The crystalline nature of the Titania NPs were also evident form the SAED pattern as shown in Fig. 2g.

### Structural, morphological and elemental analysis of Titania NPs-coated mesh membrane

Figure 3(A) depicts XRD pattern (2θ range from 20 to 90) of bare mesh and Titania NPs-coated coated mesh membrane. In Fig. 3(A), the characteristic diffraction planes (111), (200) and (220) are present for SS (stainless steel) planes in the XRD pattern of bare SS mesh. However, in the XRD pattern of Titania NPs-coated coated mesh membrane, (101), (103), (004), (112) and (200) diffraction planes pertain to the anatase phase of Titania and (110), (101) diffraction planes due to of rutile phase of Titania are present with SS planes. These figures indicate the successful deposition of Titania NPs on the surface of SS mesh.

Figure 3a–f shows the surface morphology of both bare mesh and Titania NPs-coated mesh membrane. The FE-SEM images show that bare mesh (Fig. 3a–c) has a smooth surface before coating. FE-SEM images of Titania NPs-coated mesh membrane (Fig. 3d–f) are depicting the uniform distribution of Titania NPs on the surface after the coating process. The presence of good surface roughness is also depicted in these figures. Surface roughness is a prerequisite for wettability.

**Figure 4:**
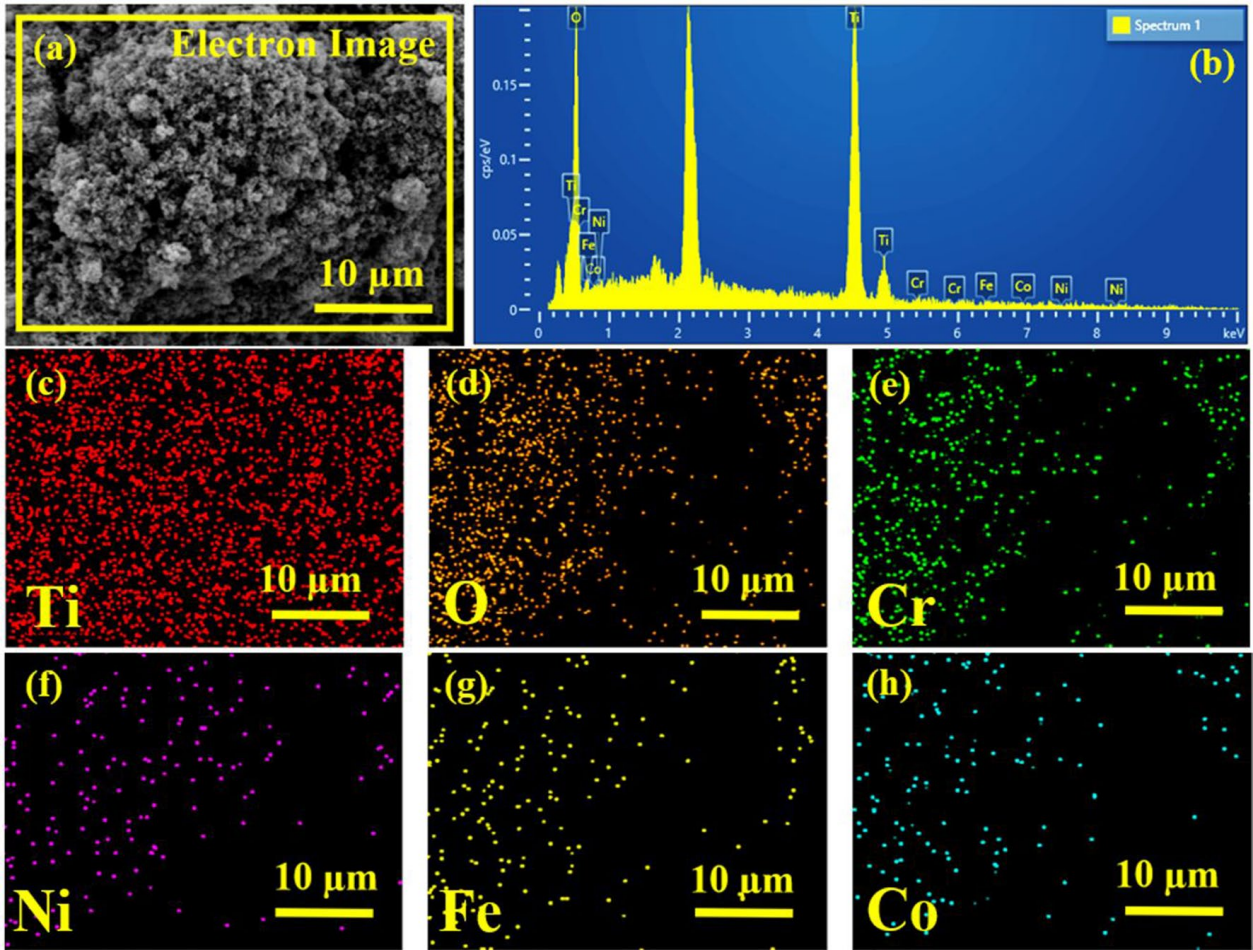
EDX analysis of Titania NPs-coated mesh membrane (**a**,**b**) and elemental mapping analysis of Titania NPs-coated mesh membrane for Ti (**c**), O (**d**), Cr (**e**), Ni (**f**), Fe (**g**), and Co (**h**).

EDX elemental analysis for Titania NPs-coated mesh membrane surface is shown in Fig. 4a,b. EDX spectrum of the Titania NPs-coated mesh membrane sample demonstrates an intense peaks of Titanium (Ti) and Oxygen (O) indicating a proper coating of Titania NPs on the surface of SS mesh (Fig. 4b). The elemental mapping images also shows that Ti and O are more dominant in the coated mesh membrane surface (Fig. 4c–h). These results clearly indicate the successful coating of Titania NPs on the surface of SS mesh. This surface was then tested for wettability under various conditions.

### Wetting/non-wetting and surface topological behavior of Titania NPs-coated mesh membrane

**Figure 5:**
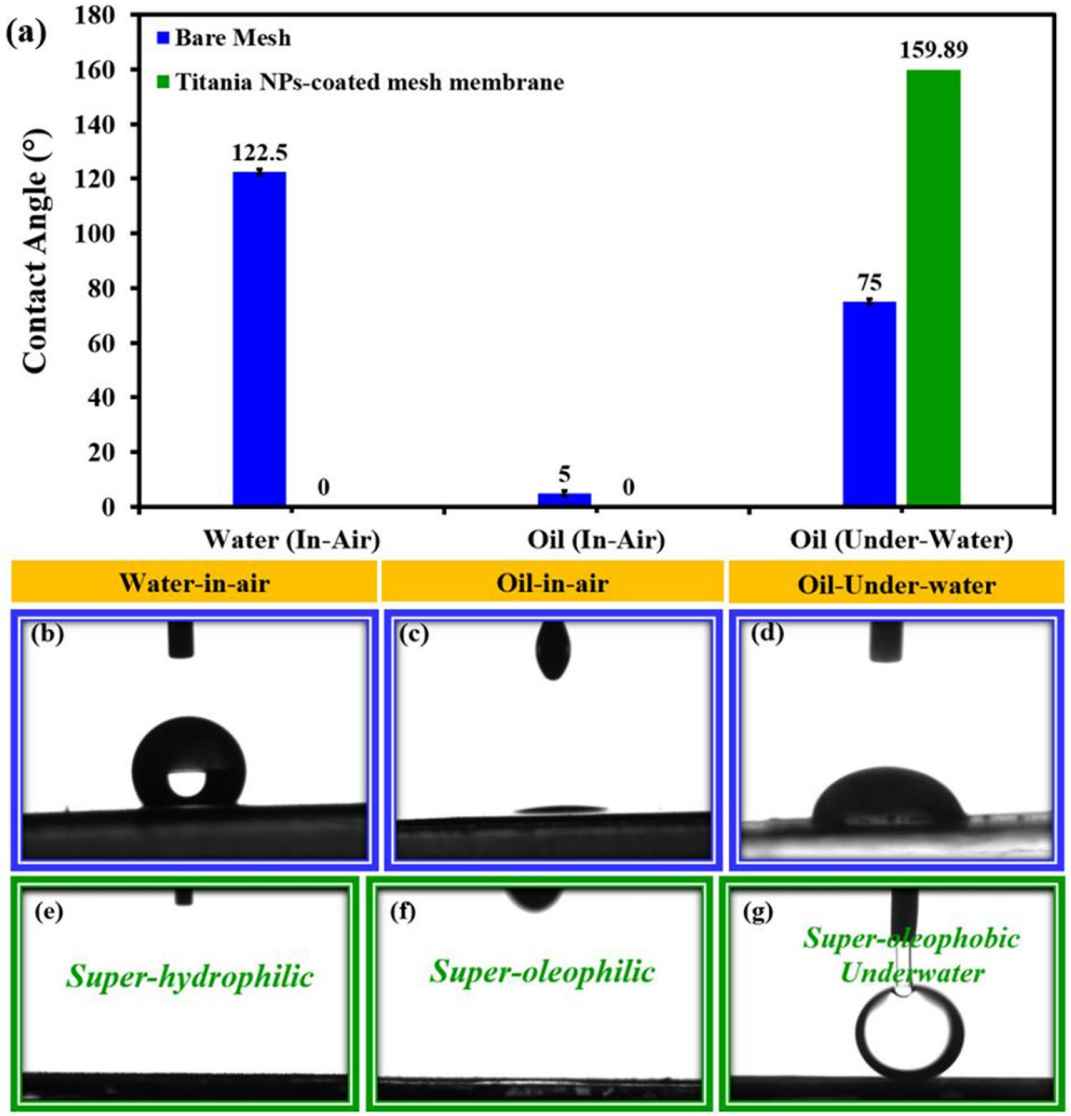
Wettability behavior for water as well as oil droplets on bare mesh and Titania NPs-coated mesh membrane surface in air and under water conditions (**a**–**g**).

The effectiveness of a membrane for oil–water separation relies on its wettability characteristics for oil and water. This is true whether the membrane is used in an air environment or under water. Key factors impacting wettability include the membrane surface roughness, its surface energy, as well as the surface tension in the case of liquids. Surface wettability can be evaluated quantitatively by determining the contact angle of liquid droplets deposited on the surface. Figure 5a shows the wettability behavior for water as well as oil droplets on bare mesh surface. The contact angle images of water in the air, oil in the air, and oil under the water on bare mesh surface are presented in Fig. 5b–d, respectively, on the uncoated mesh surface. In the air, water exhibits a contact angle of about 122.5°, while oil exhibits a contact angle of about 5°, and in the underwater environment, oil exhibits a contact angle of about 75°. These results suggest that the uncoated mesh surface is hydrophobic, super-oleophilic in the air environment, and oleophilic underwater environment. Owing to their inadequacy to separate the water and oil components of an oil–water mixture, surfaces with certain wetting characteristics are not suitable for oil–water separation.

Figure 5a also shows the wettability behavior for water as well as oil droplets on a Titania NPs-coated mesh membrane surface. The droplets are seen to fully spread across the surface in air environment, indicating contact angle approaching zero degrees. This spreading behavior of water and oil suggests the membrane possesses both super hydrophilic and super oleophilic properties in the air. This is because the surface energy of the coated membrane exceeds the surface tensions of the contacting oil and water liquids. When a solid surface energy is higher than a liquid's surface tension, it allows the liquid to fully spread and interact with the surface, yielding near-zero contact angle characterizing super wetting conditions. Therefore, Fig. 5e and f demonstrates that in air, the Titania NPs-coated mesh membrane surface preferentially wets and interacts strongly with both oil and water phases through superior oleophilicity and hydrophilicity, respectively. Figure 5g illustrates the wetting properties of oil on the Titania NPs-coated mesh membrane when submerged in water. In contrast to the spreading observed in air, oil droplets placed on the membrane under water conditions bead up into well-defined spherical caps, as evidenced by the high contact angle of approximately 160 degrees. This strong oil-repellent behavior characterizes the membrane as super oleophobic when submerged under water. Due to the membrane surface energy being lower than oil surface tension when under water, oil beads are formed.

**Figure 6:**
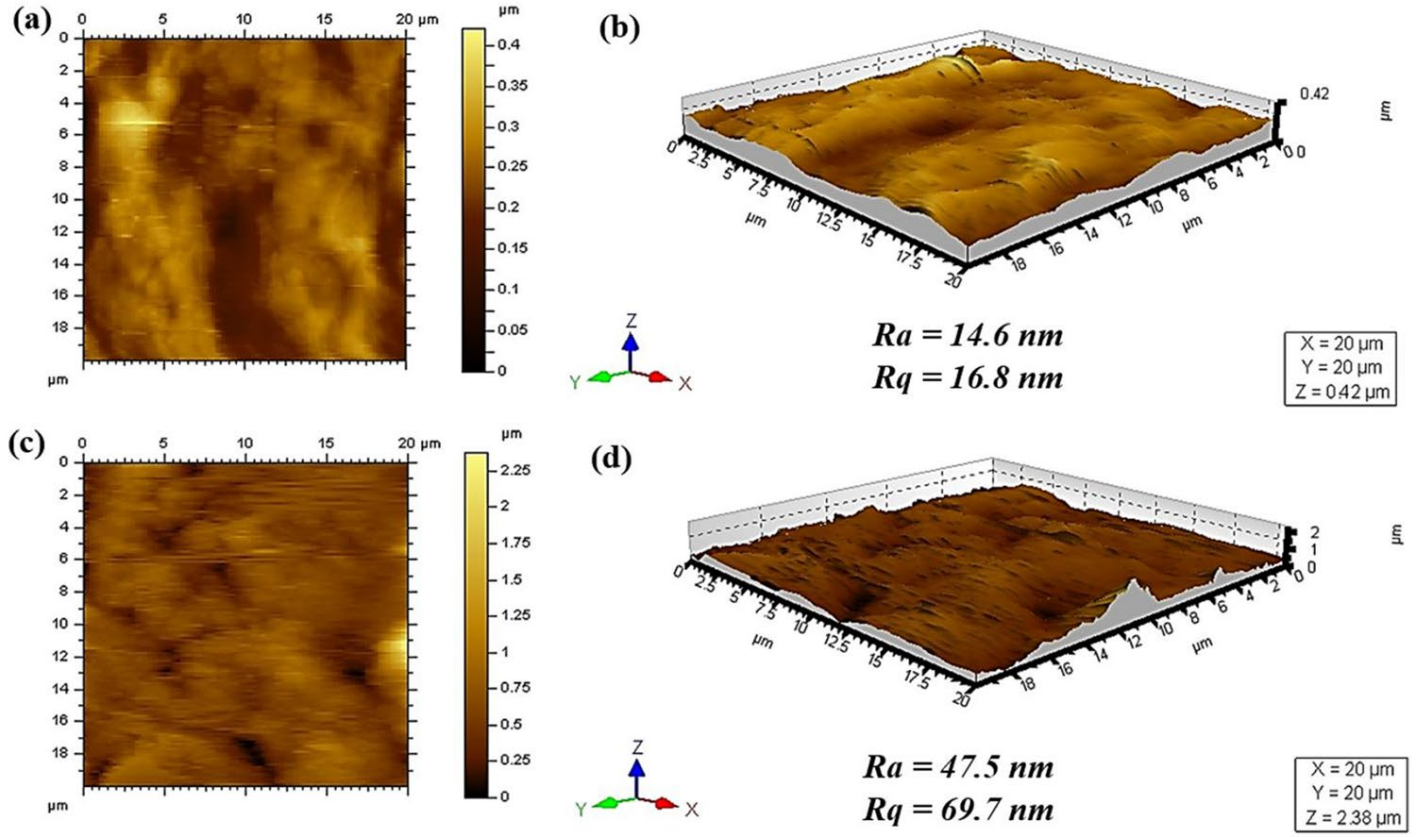
2D and 3D images of bare mesh (**a**,**b**) and Titania NPs-coated mesh membrane (**c**,**d**).

The surface roughness characteristics of the membrane likewise appear to play an important contributory role in this observed behavior^27^. The surface roughness of the Titania NPs-coated mesh membrane was evaluated and compared with that of the bare mesh in order to acquire a more thorough comprehension of the mesh membrane's surface characteristics, as illustrated in Fig. 6. It is evident from Fig. 6a and b that the bare mesh surface has smooth surface having an average surface roughness (Ra) and root-mean-square roughness (Rq) of 14.6 nm and 16.8 nm, respectively. However, after applying Titania NPs coating on the mesh surface, the Ra and Rq values of the mesh membrane coated with Titania NPs increased to 47.5 nm and 69.7 nm, respectively (Fig. 6c and d).

### Oil–water separation performance of Titania NPs-coated mesh membrane

**Figure 7:**
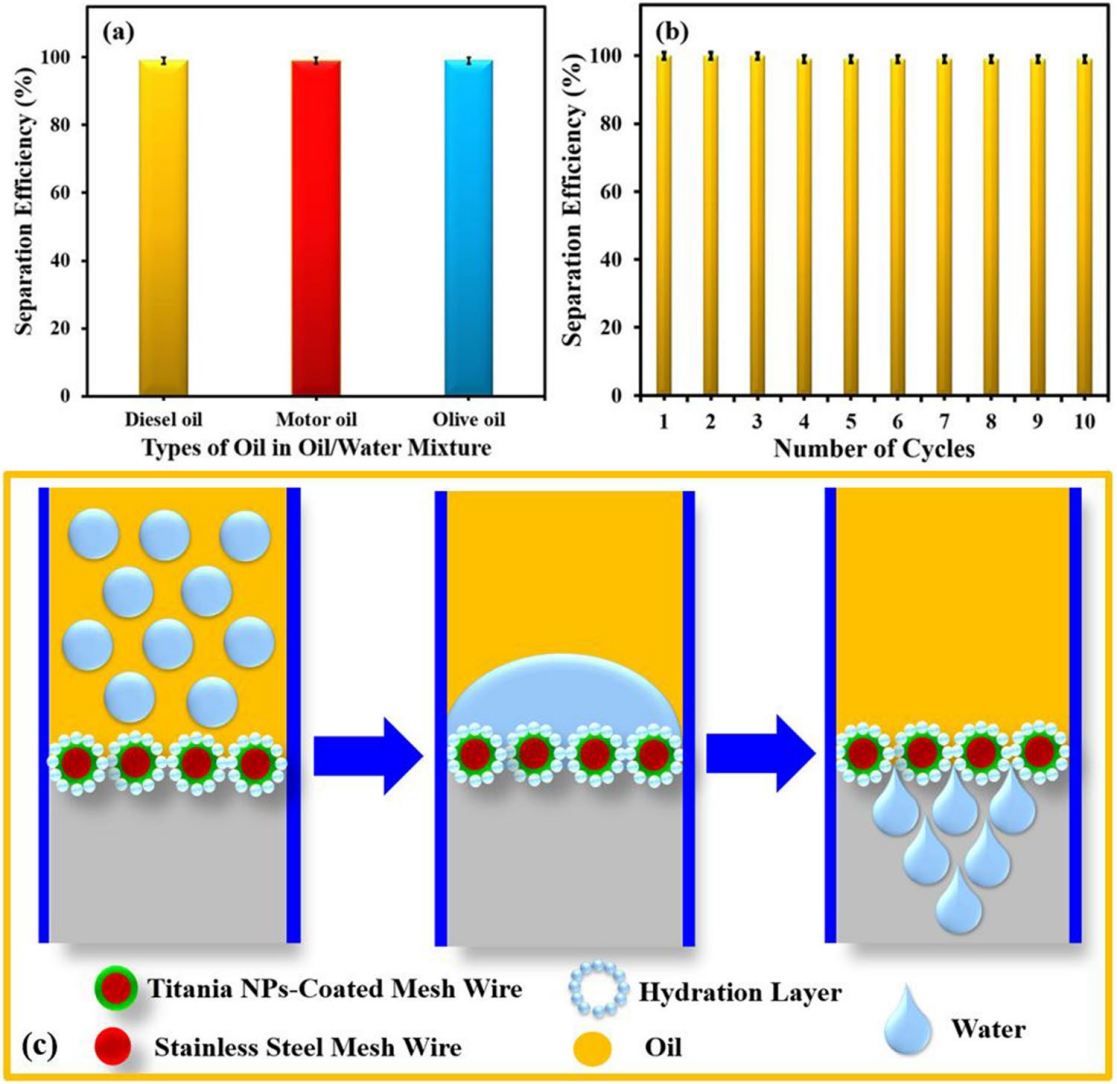
(**a**) Oil–water separation efficiency of Titania NPs-coated mesh membrane having super hydrophilic (in-air) and super oleophobic (under water) properties for different types of oil–water mixtures such as diesel oil–water, motor oil–water and olive oil–water mixture. (**b**) Stability of Titania NPs-coated mesh membrane for various oil–water separation cycles. (**c**) Graphical illustration for possible oil–water separation using Titania NPs-coated mesh membrane having super hydrophilic (in-air) and super oleophobic (under water) properties.

Oil–water separation performance of Titania NPs-coated mesh membrane having super hydrophilic (in-air) and super oleophobic (under water) properties were evaluated using lab made gravity driven filtration system for different types of oil–water mixtures such as diesel oil–water, motor oil–water and olive oil–water. In a gravity-driven oil–water separation system, the Titania NPs-coated mesh membrane with its exceptional surface wettability (super hydrophilic in-air and super oleophobic under water) demonstrated outstanding performance for oil–water separation using various oil–water mixtures as shown in Fig. 7a,b. It is evident from Fig. 7a that the separation efficiencies of Titania NPs-coated mesh membrane for all the oil–water mixtures are more than 99%. This is attributable to the membrane's dual super hydrophilic (in-air) and super oleophobic (under water) properties. Apart from the separation efficiency, the stability of the Titania NPs-coated mesh membrane was confirmed by evaluating the oil–water separation efficiency in each subsequent trial through the repeated use of the same membrane as shown in Fig. 7b. It is evident from Fig. 7b that the surface withstands ten filtration cycles without losing its typical wettability, mechanical robustness, or ability to withstand oil fouling.

### Oil–water separation mechanism

The wetting characteristics of the super-hydrophilic/super-oleophobic membrane is that when an oil–water mixture comes in contact with membrane under the water, the water phase is attracted and wets the membrane while the oil beads up and gets repelled, paving the way for effective oil water separation as in Fig. 5g. As previously stated, the amount of water or oil that wets the surface depends on the relative strengths of the liquid's surface tension and the membrane's surface energy as well as the surface's hierarchical roughness factor. In contrast, when the surface energy of the membrane is less than the surface tension of the liquid, the adhesive force between the liquid and the surface is less than the cohesive force of the liquid, resulting in the repulsion of liquid on the surface. This phenomenon occurs when the surface energy of the membrane is higher than the surface tension of the liquid. By surface proper engineering by incorporating appropriate surface roughness, composition and surface energy the surface wettability for oil and water can be optimized. This targeted wettability then determines the functionality of a membrane and its ability to separate oil from water effectively. One of the active areas of research is to investigate ways to engineer new membrane designs with targeted controlled wetting properties in order to maximize their separation performance. The degree of wettability is experimentally measured based on the contact angle that liquids make when placed on solid surfaces in air or under the water ambiance.

Figure 5e and f displays images of droplets of water and oil on the Titania NPs-coated mesh membrane in different environments, where particularly the spreading and beading of oil droplet on the membrane surface respectively in air and under the water are visually evident. The figure also shows that the total spreading of oil or water on the surface leads to near zero contact angle, whereas the beading of oil under the water gives high contact angle. From the visual examinations and the contact angle measurements it can be concluded that the membrane surface is both super-hydrophilic and super-oleophilic in the air environment. The observed surface wettability of oil and water in air environment is due to the fact that the surface energy of Titania NPs-coated mesh membrane is greater than the surface tension of both oil and water, leading to the strong adhesion of oil and water on the membrane surface. On the other hand, when the oil droplets come in contact with the membrane under the water, the oil beads on the surface, and this is because the trapped water in the pits of the membrane prevents the oil to have direct interaction with the surface, rather the oil droplets hovers on the water surface. This under water wetting behavior of the oil on the surface resulted in oil contact angle (under water) as high as 160°. When submerged in water, the highly hydrophilic nature of the membrane causes a film of water to form on its surface as shown in Fig. 5g. With a lowered surface energy beneath the water, the membrane exhibits high level of oleophobicity according to the basic Young’s law applied to oil-surface-air interface. The switching of surface wettability of membrane from super oil-affinity in air to super oil-repulsion is the key for the effective applicability of this kind of membrane for oil water separation. Observations of the time line of the wetting behavior of oil and water droplets on the surface in air compared to underwater is notably different as shown in Fig. 7c, where in air both oil and water droplets rapidly spread across the entire surface within a short time period, unlike, the oil droplets which remained beaded shape for a much longer duration. In reality, Young’s model of surface wettability failed to explain the discrepancy between the experimental contact angle and the contact angle predicted by Young’s equation^28^. This anomaly was removed by the modified surface wettability models proposed by Wenzel and Cassie Baxter, both of which incorporated the surface roughness in the Young’s equation to account for the experimental contact angle in non-ideal surface. The in air superhydrophilcity and underwater superoleophobicity in Titania NPs-coated mesh membrane was brought about by the combination of the surface energy and the hieratical surface roughness. This combination of superhydrophilicity (attracting water) and superoleophobicity (repelling oil) allows the membrane to effectively separate the two immiscible phases upon contact with an oil–water mixture (Fig. 7c).

### Environmental impacts using life cycle assessment

The results of LCA for preparing mesh membranes are given in Table 2. The en-vironmental impacts are expressed using ReCiPe midpoint categories^6^. The impact categories along with units are also listed in Table 2. The results show that the produc-tion of 6 cm × 6 cm Titania NPs-coated mesh membrane has a global warming potential of 0.24 kg of CO_2_, terrestrial acidification potential equal to 0.001 kg of SO_2_, and human carcinogenic toxicity equal to 0.003 kg of 1,4–DCB. As the process industrialized, these values can increase significantly.

**Table 2:** LCA results.

Impact category	Symbol	Unit	Impact value
Global warming	GW	kg CO_2_ eq	0.2397615
Stratospheric ozone depletion	OD	kg CFC-11 eq	1.2224E-07
Ionizing radiation	IR	kBq Co-60 eq	0.0053089
Ozone formation, human health	OFH	kg NOx eq	0.0005946
Fine particulate matter formation	FPMF	kg PM2.5 eq	0.0003868
Ozone formation, terrestrial ecosystems	OFT	kg NOx eq	0.0006093
Terrestrial acidification	TA	kg SO_2_ eq	0.0010171
Freshwater eutrophication	FE	kg P eq	2.8452E-05
Marine eutrophication	ME	kg N eq	2.1942E-06
Terrestrial ecotoxicity	TE	kg 1,4-DCB	0.5087561
Freshwater ecotoxicity	FET	kg 1,4-DCB	0.0023239
Marine ecotoxicity	MET	kg 1,4-DCB	0.0033713
Human carcinogenic toxicity	HCT	kg 1,4-DCB	0.0030706
Human non-carcinogenic toxicity	HCNT	kg 1,4-DCB	0.0623332
Land use	LU	m^2^a crop eq	0.0083271
Mineral resource scarcity	MRS	kg Cu eq	0.0003277
Fossil resource scarcity	FRS	kg oil eq	0.0769528
Water consumption	WC	m^3^	0.0017686

**Figure 8:**
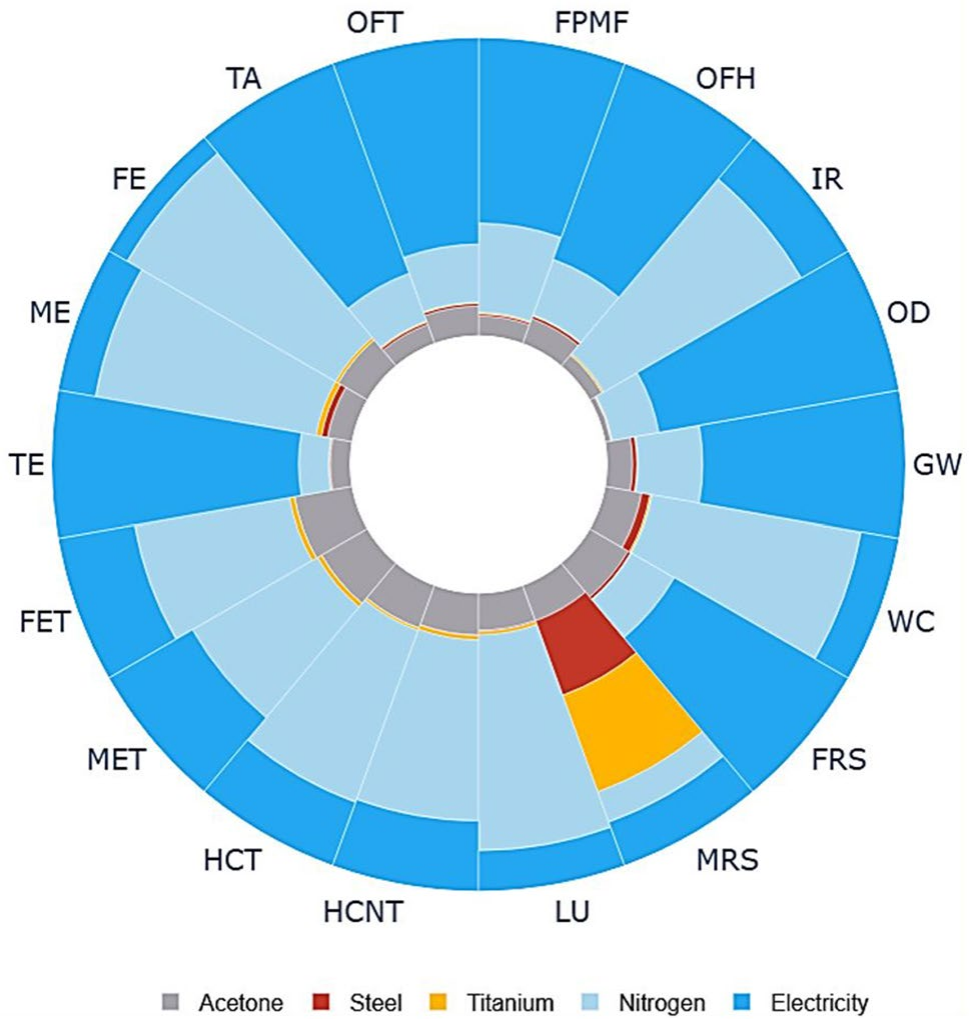
Normalized cradle-to-gate environmental profile.

To evaluate the contribution of different inputs of the process to environmental impacts, a normalized plot is shown in Fig. 8. In this figure, each environmental impact is normalized and the total value for each category is 100%. As it can be seen from the plot electricity and nitrogen gas are the main contributors towards all environmental impact categories. For instance, electricity used for ovens contributes 68% towards global warming, 81% towards ozone depletion, and 74% towards terrestrial acidification. Similarly, the use of nitrogen gas is the major contributor to ionizing ra-diation (76%), freshwater eutrophication (80%), and water consumption (71%). These findings show that electricity and nitrogen are the environmental hotspots in the fabrication of Titania NPs-coated mesh membrane. In future studies, design of experiments approach should be used to optimize electricity and nitrogen consumption during the coating process. Design of experiments will help in accurately representing relationships between coating process variables, nitrogen and electricity consumption, and process performance. Also, regional variations in electricity generation should also be considered. Using this approach will lead to optimal use of electricity and nitrogen thus reducing both environmental impacts and cost of the process.

## Conclusions

Under-water super oleophobic and in-air super hydrophilic Titania NPs-coated mesh membrane was manufactured by spray coating of Titania NPs on SS mesh and then calcining the membrane at 500 °C, and the life cycle assessment (LCA) was used in order to evaluate the environmental impacts of the Titania NPs-coated mesh membrane fabrication process for the oil remediation application. XRD and FE-SEM analysis of the membrane showed successful coating of Titania NPs on the surface of SS mesh. The contact angle measured using a goniometer showed that the contact angle for oil (under water) is close to 160° (super-oleophobic) and for water (in-air) is 0° (super hydrophilic). This suggests that the Titania NPs-coated mesh membrane could be employed in the gravity-driven oil–water separation system as a separation medium. The results of the life cycle analysis demonstrate that both nitrogen and electricity used in the coating process contributed the most toward 18 environmental impact categories. This LCA adds new knowledge to the production of mesh membranes through coating and it can help in mitigating the environmental impacts of the process.

## Data Availability

All data generated or analyzed during this study are included in this published article.
