# Author Correction: Development and life cycle assessment (LCA) of super-oleophobic (under water) and super-hydrophilic (in-air) mesh membrane for oily water treatment

**DOI:** 10.1038/s41598-025-24147-9

**Published:** 2025-10-31

**Authors:** Umair Baig, M. Mobeen Shaukat, S. Z. Shuja, M. Asif, Nadeem A. Khan

**Affiliations:** 1 Interdisciplinary Research Center for Membranes and Water Security, King Fahd University of Petroleum and Minerals, 31261 Dhahran, Saudi Arabia; 2 Department of Mechanical Engineering, King Fahd University of Petroleum and Minerals, 31261 Dhahran, Saudi Arabia; 3 Interdisciplinary Research Center for Renewable Energy and Power Systems, King Fahd University of Petroleum and Minerals, 31261 Dhahran, Saudi Arabia; 4 Department of Architectural Engineering, King Fahd University of Petroleum and Minerals, 31261 Dhahran, Saudi Arabia

Correction to: *Scientific Reports* 10.1038/s41598-024-64803-0, published online 03 July 2024

The original version of the Article contained an error by omission, where a contextually relevant article was not cited. The below Reference has been added and is listed as Reference 19.

19. Baig, U. & Dastageer, M. A. Fabrication of photo-responsive mesh membrane with surface-engineered wettability for oil–water separation and photocatalytic degradation of organic pollutants. *Membranes* 13(3):302 (2023)

As a result of the changes, the References have been renumbered.

Additionally, in the Introduction section,

“In this study, Titania (TiO_2_) nanoparticles (NPs) were chosen as a coating material for the fabrication of super-oleophobic (under water) and super-hydrophilic (in-air) mesh membrane due to its exceptional super-hydrophilicity, high stability, cheap cost, and commercial availability.”

now reads:

“In this study, based on our previous findings^19^, Titania (TiO_2_) nanoparticles (NPs) were chosen as a coating material for the fabrication of super-oleophobic (under water) and super-hydrophilic (in-air) mesh membrane due to its exceptional super-hydrophilicity, high stability, cheap cost, and commercial availability, in order to determine the environmental impacts of Titania NPs-coated mesh membrane fabrication process using life cycle assessment (LCA).”

In the Experimental methods section, under the subheading “Mesh membrane fabrication and characterization”,

“The mesh (stainless-steel; SS) used for this study was acquired from TWP Inc USA and Titania NPs (particle size 10–20 nm) were acquired from Sigma Aldrich (USA).”

now reads:

“The Titania NPs-coated mesh membrane was fabricated by the same method as described in our previous study^19^. The mesh (stainless-steel; SS) used for this study was acquired from TWP Inc USA and Titania NPs (particle size 10-20 nm) were acquired from Sigma Aldrich (USA).”

Finally, in the Conclusion section,

“Under-water super oleophobic and in-air super hydrophilic Titania NPs-coated mesh membrane was manufactured by spray coating of Titania NPs on SS mesh and then calcining the membrane at 500 °C.”

now reads:

“Under-water super oleophobic and in-air super hydrophilic Titania NPs-coated mesh membrane was manufactured by spray coating of Titania NPs on SS mesh and then calcining the membrane at 500 °C, and the life cycle assessment (LCA) was used in order to evaluate the environmental impacts of the Titania NPs-coated mesh membrane fabrication process for the oil remediation application.”

The original Article has been corrected.

